# Identification of regulators of the myofibroblast phenotype of primary dermal fibroblasts from early diffuse systemic sclerosis patients

**DOI:** 10.1038/s41598-019-41153-w

**Published:** 2019-03-14

**Authors:** Loubna Chadli, Britt Sotthewes, Kejie Li, Stefan N. Andersen, Ellen Cahir-McFarland, Marc Cheung, Patrick Cullen, Annemarie Dorjée, Jeska K. de Vries-Bouwstra, Tom W. J. Huizinga, David F. Fischer, Jeroen DeGroot, Joanne L. Viney, Timothy S. Zheng, Jamil Aarbiou, Agnes Gardet

**Affiliations:** 1 Charles River Nederland BV, Leiden, The Netherlands; 2 Biogen, Cambridge, MA USA; 3 Department of Rheumatology, Leiden University Medical Center, Leiden, The Netherlands

## Abstract

Systemic sclerosis (SSc or scleroderma) is an auto-immune disease characterized by skin fibrosis. While primary cells from patients are considered as a unique resource to better understand human disease biology, the effect of *in vitro* culture on these cells and their evaluation as a platform to identify disease regulators remain poorly characterized. The goal of our studies was to provide insights into the utility of SSc dermal fibroblast primary cells for therapeutic target discovery. The disease phenotypes of freshly isolated and *in vitr*o cultured SSc dermal fibroblasts were characterized using whole transcriptome profiling, alpha smooth muscle actin (ASMA) expression and cell impedance. SSc dermal fibroblasts retained most of the molecular disease phenotype upon *in vitro* culture for at least four cell culture passages (approximatively 10 cell doublings). We validated an RNA interference high throughput assay that successfully identified genes affecting the myofibroblast phenotype of SSc skin fibroblasts. These genes included *MKL1*, *RHOA* and *LOXL2 that* were previously proposed as therapeutic anti-fibrotic target, and *ITGA5*, that has been less studied in fibrosis biology and may be a novel potential modifier of SSc fibroblast biology. Together our results demonstrated the value of carefully-phenotyped SSc dermal fibroblasts as a platform for SSc target and drug discovery.

## Introduction

Systemic sclerosis (SSc) is a chronic auto-immune disease associated with high mortality and high morbidity^1^. Initially, the organ most strongly affected is the skin, but the fibrotic process can progress to other organs such as the respiratory system and the gastro-intestinal tract. Both inflammatory and fibrotic processes are thought to contribute to the disease pathogenesis^2^. Among the disease effector cells, activated dermal fibroblasts or myofibroblasts are considered to play a major role in the development of the skin fibrosis, due to their ability to produce large amounts of extracellular matrix and support tissue contractility^3^. Indeed, analyses of SSc skin biopsies have shown that the increase of myofibroblasts and large collagen fibrils correlates with modified Rodnan Skin Score (mRSS), which is a clinical score to estimate skin thickness^4,5^. Whole transcriptome analyses of skin biopsies from SSc patients also highlighted fibroinflammatory signatures; with a prominent fibroblast signature and an increase of the expression of extracellular matrix transcripts^6–10^. Pathway analysis using these transcriptional data suggested that the TGFβ, PDGF, WNT and the interferon pathways may be involved in the abnormal skin transcriptome of SSc patients^11–13^. Among them, the TGFβ pathway is thought to play a critical role in the disease pathogenesis, as it acts as a potent inducer of fibroblast activation and extracellular matrix production^13,14^.

Similar to the findings from studies with SSc skin biopsies, cultured primary SSc dermal fibroblasts also appear to show a profibrotic phenotype at the transcriptional level^7,15,16^. They produce increased amounts of extracellular matrix, including collagen 1 and fibrillin^17,18^ and they express higher levels of SMAD3 and TGFβ-activating integrins, such as αv, β5 and β1, supporting the relevance of the TGFβ pathway for their abnormal phenotype^19–21^. In addition, a proportion of dermal SSc fibroblasts seem to be differentiated into myofibroblasts, with an increased expression of CTGF and ASMA as well as a more pronounced contractile phenotype, which is thought to contribute to the stiffness of the fibrotic tissue^21–23^. Besides their profibrotic nature, SSc dermal fibroblasts have also been proposed to contribute to the inflammation in the SSc skin as they secrete higher amounts of IL-6 compared to dermal fibroblasts isolated from healthy donors at baseline or in response to proinflammatory stimulations, such as TFNα or a combination of TNFα and IFNγ^24,25^. Together, the discoveries of these abnormal SSc fibroblast phenotypes motivated multiple efforts to identify modifiers of fibroblast biology as an anti-fibrotic strategy, including targeting the TGFβ pathway, the extracellular matrix organization and myofibroblast contractility^26^.

Human primary fibroblasts isolated from various organs are often used to support fibroblast biology studies and multiple potential fibroblast therapeutic targets have emerged from these efforts. Although characterization and functional studies have highlighted the abnormal behavior of SSc dermal fibroblasts, there are still limited reports on how potential disease modifiers may affect primary SSc fibroblast abnormal biology likely due to the difficult access to the primary cells^22,23,27^. In this study, we aimed to investigate whether human primary SSc dermal fibroblasts could be used as a platform to support target identification of regulators of SSc disease phenotype and whether they would be suitable for RNA interference comprehensive screens. Thus, we characterized the disease phenotypes of dermal fibroblasts isolated from SSc skin biopsies using transcript profiling, ASMA expression as a myofibroblast marker, and cell impedance as a surrogate for cell morphology differences. Using an adenoviral shRNA platform, we confirmed that a high throughput RNAi screen could be achieved with human primary SSc dermal fibroblasts with adequate robustness and throughput (384-well format). We successfully identified top regulators of ASMA expression in primary SSc dermal fibroblasts among a set of 87 genes enriched for genes relevant to myofibroblast biology. Overall, this study highlights the need to carefully understand and define the disease phenotypes of primary disease cells and how these phenotypes are maintained in *in vitro* system in order to assess the value patient primary cells for target discovery and drug discovery.

## Results

### Fibroblasts isolated from SSc skin biopsies retain part of SSc transcriptional signature up to at least four *in vitro* culture passages

Skin biopsies were obtained from 10 healthy donors and from 6 donors affected by early diffuse SSc from clinically affected or non-affected skin area (Table 1 provides a summary of the characteristics of the donors, Supplementary Table 1 provides the information on what data were collected for each donor). Microarray analyses revealed that skin biopsies from SSc donors showed different transcript profiles than skin biopsies obtained from healthy donors. Principal component analysis confirmed that SSc samples clustered separately from healthy samples (Supplementary Fig. 1A). There were 1178 probes differentially expressed between the SSc skin biopsies and the healthy skin biopsies (Supplementary Fig. 1B and Table 2). Pathway analysis revealed that SSc differentially expressed genes were enriched in genes involved in extracellular matrix organization and immune pathways as well as an interferon signature previously associated with SSc skin (Supplementary Fig. 1C–E). Within the SSc samples, the skin biopsies obtained from disease affected skin area could not clearly be differentiated from the ones obtained from non-affected skin area as shown by the principal component analysis (Supplementary Fig. 1A). Only 2 transcripts were detected to be statistically differentially expressed with a lower expression in biopsies obtained from affected site vs non-affected site (HOXB-AS3, HOXB7). This was consistent with the previous studies reporting the challenges of identifying differences at the transcriptional levels between SSc skin biopsies obtained from clinically affected site vs non-affected skin area^7,9,28,29^. Overall, microarray transcriptomic analyses confirmed that the skin biopsies that were used to isolate the SSc primary fibroblasts recapitulated the disease signatures previously described by various groups^6–10,28^.

**Table 1:** Characteristics of the Donors.

Criteria\Donor	SSc01	SSc02	SSc03	SSc04	SSc05	SSc06	SSc07	SSc08	HS (n = 11)
Gender	F	F	F	M	M	F	F	F	F
Ethnicity	Caucasian	Caucasian	Caucasian	Caucasian	Caucasian	Caucasian	Caucasian	Caucasian	Caucasian (10/11)
Patient age	37	63	81	54	48	54	39	50	47+/−9.5
diffuse cutaneous SSc	Yes	Yes	Yes	Yes	Yes	Yes	Yes	Yes	NA
Disease Duration (months)	11	14	5	2	4	4	4	2	NA
First non-Raynaud symptoms (months prior skin collection)	11	15	5	7	4	10	4	6	NA
Raynaud manifestations	Yes	Yes	Yes	Yes	Yes	Yes	Yes	Yes	NA
Modified Rodnan skin score	7/51	31/51	13/51	29/51	37/51	14/51	21/51	18/51	NA
Auto-antibodies	PmScl	fibrillarin/U3 RNP	Topoisomerase (Scl-70)	Topoisomerase (Scl-70)	Topoisomerase (Scl-70)	Topoisomerase (Scl-70)	fibrillarin	RNA pol III	NA
Treatment	Hydroxychloroquine Prednisone Methotrexate	No immuno-suppressive therapy	No immuno-suppressive therapy	No immuno-suppressive therapy	Methotrexate (3 wks)	No immuno-suppressive therapy	Methotrexate (2 months)	Methotrexate (day of biopsy collection)	NA

**Figure 1:**
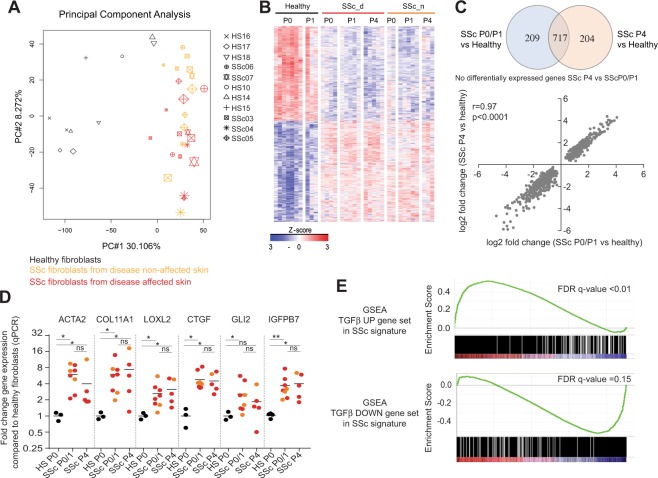
Microarray gene expression analyses of freshly isolated and *in vitro* cultured primary SSc dermal fibroblasts. Microarray gene expression data from fibroblasts from Passage 0 to Passage 4 from 5 SSc patients (isolated from disease affected skin (SSc_d) or non-disease affected skin (SSc_n)) and 7 healthy donors were analyzed. (**A**) Principal Component Analysis. (**B**) Z-score heatmap showing the gene expression profiles of the differentially expressed probes between SSc dermal fibroblasts at P0/P1 and healthy dermal fibroblasts at P0/P1. (**C**) Overlap of the differentially expressed genes from SSc dermal fibroblasts P0/P1 compared to healthy dermal fibroblasts and from SSc dermal fibroblasts P4 compared to healthy dermal fibroblasts. (**D**) Quantitative PCR validation data from fibroblasts from at least 5 SSc patients isolated from non-affected skin (orange) or affected skin (red) and 3 healthy donors (black). Statistical significance was assessed using Mann-Whitney test with *p < 0.05 and **p < 0.01. (**E**) Gene Set Enrichment Analysis of a published *in vitro* TGFβ gene signature in the SSc signatureTop panel shows a Venn diagram showing the number of unique and overlapping differentially expressed genes. Bottom panel is a plot showing correlation between the log2 fold changes of the union of the differentially expressed genes.

Dermal fibroblasts were isolated from skin biopsies from SSc patients and healthy individuals and cultured up to four cell culture passages, which corresponds approximatively to 10 cell doublings from the freshly isolated. Two microarray experiments were conducted to analyze the transcriptomes of these primary cells at different passages. Since the microarray data were collected from 2 different experiments, data were first subjected to batch correction before differential gene expression analyses (Supplementary Fig. 2). The microarray gene expression data could differentiate freshly isolated SSc skin fibroblasts from healthy skin fibroblasts with the identification of 926 differentially expressed unique genes (1365 differentially expressed probes) between SSc skin fibroblasts (passage 0 and passage 1, P0/P1) and healthy skin fibroblasts (Passage 0 and 1, P0/P1) (Fig. 1A–C and Supplementary Table 3). Both principal component analysis and a heatmap showing the gene expression profiles of the SSc fibroblast signature highlight that SSc skin fibroblasts from passage 0 (P0) to passage 4 (P4) showed very similar gene expression profiles (Fig. 1A,B). No transcript passed the 1.5-fold change threshold and FDR-adjusted p-value of less than 0.01 between when directly comparing SSc P4 and P0P1 groups (Supplementary Table 4). The SSc skin fibroblasts showed upregulation of the typical fibrotic genes such as components of the extracellular matrix (e.g. collagens, tenascin, decorin, lumican, aggrecan transcripts and metalloproteases). Quantitative PCR data confirmed the upregulation in SSc fibroblasts of several transcripts of genes known to be associated with fibrosis and myofibroblast biology and detected in our microarray analysis: *ACTA2* (encoding for ASMA), extracellular matrix associated genes (*COL11A1*, *LOXL2*, *CTGF*), a known myofibroblast transcription factor (*GLI2*) and a known profibrotic secreted factor (*IGFBP7*) (Fig. 1D)^30–32^. In addition, we also detected the enrichment for an *in vitro* TGFβ gene expression signature (Fig. 1E)^33^. SSc skin fibroblasts cultured for up to four passages (P4) were transcriptionally similar to freshly isolated SSc skin fibroblast (P0/P1) (Fig. 1A,B). Out of the 926 differentially expressed probes detected at P0/P1 between SSc and healthy fibroblasts, 717 of them were remained differentially expressed at P4 (Fig. 1C and Supplementary Table 3). There was a strong correlation between the fold changes of the differentially expressed genes between SSc P0/P1 or SScP4 vs healthy fibroblasts (Fig. 1C). Similar to what was observed with the skin biopsies, transcriptional analyses could not differentiate SSc skin fibroblasts obtained from biopsies from clinically affected skin area vs clinically non-affected skin area (Fig. 1A,B). No transcript passed the 1.5-fold change threshold and FDR-adjusted p-value of less than 0.01 between fibroblasts from clinically affected skin area vs non-affected skin area at passage 0 (Supplementary Table 5).

In summary, the microarray gene expression analyses confirmed that the SSc skin fibroblasts isolated from patients’ skin biopsies largely preserved the disease transcriptional SSc disease signature up to at least four *in vitro* cell culture passages.

### Fibroblasts isolated from SSc skin biopsies show a higher alpha smooth muscle actin expression level for up to four *in vitro* culture passages compared to fibroblasts isolated from healthy skin

**Figure 2:**
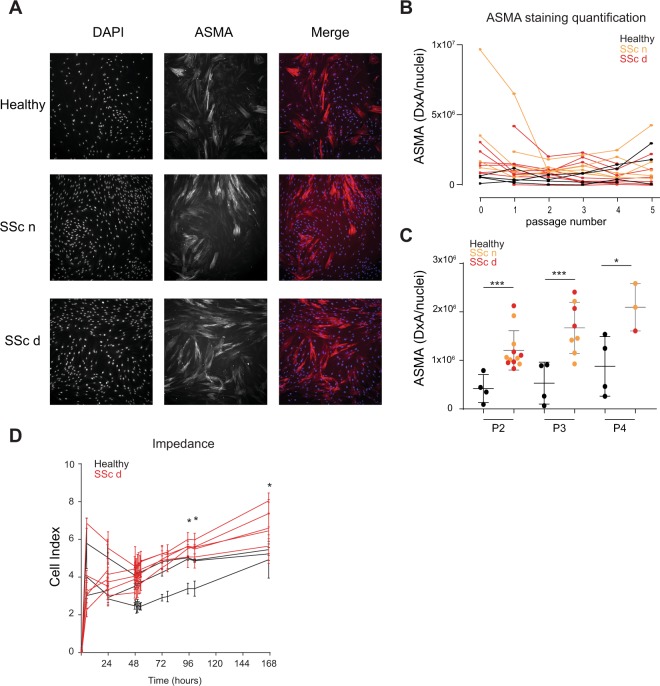
High ASMA expression and abnormal cell impedance are detected in primary SSc dermal fibroblasts up to four *in vitro* culture passages. Primary dermal fibroblasts from SSc donors and healthy donors were immuno-stained to measure ASMA expression and stained with DAPI to detect nuclei. (**A**) Representative images of the ASMA and DAPI stainings from dermal fibroblasts isolated from a healthy donor and an SSc donor. The SSc dermal fibroblasts were isolated from a clinically affected area (SSc d) and non-affected area (SSc n). (**B**) Image quantification results of ASMA staining (Density X Area corresponding to the intensity of the staining multiplied by the area) divided by the number of nuclei stained with DAPI of SSc dermal fibroblasts (orange and red) or healthy dermal fibroblasts (black) at different cell culture passages. (**C**) Differences in ASMA staining (Density X area) divided by the number of nuclei between SSc dermal fibroblasts and healthy dermal fibroblasts at different cell culture passages. (**D**) Cell index from SSc dermal fibroblasts and healthy dermal fibroblasts after four *in vitro* cell passage. Statistical significances were assessed using two-tailed Student’s t-tests with *p-value < 0.05, ***p-value < 0.001.

Fibroblasts isolated from chronic fibrotic tissues are known to typically express higher alpha smooth muscle actin (ASMA) levels leading to an increased contractility and cellular morphological change. Using ASMA immunofluorescent staining, we confirmed that overall SSc dermal fibroblast expressed a higher amount of ASMA protein than healthy dermal fibroblasts for up to four *in vitro* cell culture passages (Fig. 2A,B). At passage 2, SSc fibroblasts from 7 donors out of 7, either isolated from clinically affected skin or non-affected skin, showed higher ASMA expression levels than healthy dermal fibroblasts from 4 donors at passage 2 (Fig. 2C). At passage 3, SSc fibroblasts from 7 donors out of 7 donors (5 cultures isolated from non-affected area and 3 from affected area) showed higher ASMA expression levels than healthy dermal fibroblasts (Fig. 2C). At passage 4, SSc fibroblasts from 3 donors out of 7 (2 lines isolated from non-affected area and 1 from affected area) showed higher ASMA expression levels than healthy dermal fibroblasts from 4 donors (Fig. 2C). Noteworthy, *in vitro* culture led to an overall increase of ASMA levels in healthy fibroblasts likely due to the time of culture on plastic cell culture flasks, which present as a matrix with high stiffness (Fig. 2C). Consistent with a higher expression of ASMA and functional effect of cell cytoskeleton, the impedance readout of monolayers of SSc dermal fibroblasts showed statistical difference compared to the impedance measured from healthy dermal fibroblasts after four *in vitro* cell culture passages (Fig. 2D).

Cell expansion is necessary to obtain sufficient number of cells for functional studies. Together our data showed that expanded SSc dermal fibroblasts up to four *in vitro* cell culture passages from multiple donors maintained about 77% disease transcriptional signature compared to freshly isolated disease cells, in addition to a high ASMA expression level and abnormal cell impedance. This suggests that SSc dermal fibroblasts may be used as a platform to identify regulators of disease fibroblast phenotypes after a limited cell culture expansion.

### Adenovirus delivery of shRNAs in SSc dermal fibroblasts leads to efficient gene expression knockdown and enables to identify regulators of ASMA expression in SSc dermal fibroblasts

**Figure 3:**
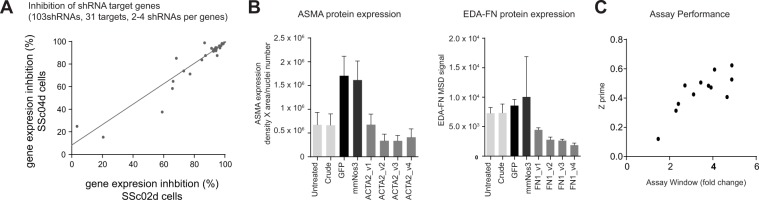
Transduction of shRNAs using Adenovirus leads to efficient gene expression knockdown in primary SSc dermal fibroblasts. (**A**) Gene expression knockdown after shRNA transductions of SSc dermal fibroblasts. Plot shows the percentage of inhibition in gene expression for each shRNA in dermal fibroblasts from two different SSc donors (**B**) SSc dermal fibroblasts were transduced by shRNAs targeting GFP and mmNOS3 (negative controls) as well as 4 different shRNAs targeting *ACTA2* or *FN1*. Crude conditions used only incubation with cell lysates from PerC5 cells that were used to produce the adenovirus. ASMA protein expression was measured using ASMA immunostaining after image quantification and normalization with the number of nuclei. EDA-Fibronectin protein expression was measured using an MSD assay. (**C**) Twelve SSc dermal fibroblasts (obtained from disease-affected skin or non-disease affected skin from 6 different SSc donors) were transduced with shRNAs targeting GFP (negative control) or ACTA2. Differences in ASMA protein expression between the GFP shRNA and the ACTA2 shRNA were determined using ASMA immunostaining after image quantification and normalization with the number of nuclei. Plot show both Z-primes and assay windows for each SSc dermal fibroblast tested (based on 120 data points for each donor and each shRNA).

Using adenoviral delivery system, shRNAs were transduced into SSc primary fibroblasts. Gene expression knockdown was assessed with quantitative PCR after transduction of 103 shRNAs targeting 31 different genes with 2–4 shRNAs per gene into dermal fibroblasts from 2 different SSc donors. In cells from both donors, approximatively 80% of shRNAs led to more than 60% target gene expression knockdown and approximatively 65% of shRNAs led to more than 75% target gene expression knockdown (Fig. 3A). This demonstrated that the adenovirus delivery of shRNAs was an efficient RNAi platform for primary SSc dermal fibroblasts. Protein knockdown was also successfully achieved after transduction of shRNAs targeting either ASMA or fibronectin, as assessed using ASMA immunofluorescence staining or EDA-Fibronectin MSD assay (Fig. 3B).

Using ASMA immunofluorescent staining data from SSc dermal fibroblasts isolated from clinically affected or non-affected skin area from 6 different donors and transduced with shRNAs targeting either ASMA or GFP (negative control), we evaluated how well the assay could measure inhibition of ASMA expression. Based on 120 data points for each donor and each shRNA, the difference in ASMA expression level from the ASMA shRNA conditions showed that the assay window was consistently 2–4 fold and the assay Z-prime values were mostly between 0.3–0.6 (Fig. 3C), demonstrating this assay would be robust for high throughput screening to identify inhibitors of ASMA expression in SSc dermal fibroblasts.

Noteworthy, there were two observations suggesting that, although the assay had the potential to identify regulators of the myofibroblast phenotype of SSc cells, the assay conditions did alter the SSc dermal fibroblasts. First, the viral transduction seemed to induce an increase of ASMA protein expression of SSc dermal fibroblasts, but not FN1 (Fig. 3B). Second, an additional transcriptomic analysis using cells at time of the ASMA assay readout highlighted that part of the SSc disease signature was affected by the 7-day cell culture condition, which contained low fetal bovine serum (0.5% vs 10% FBS in the expansion culture medium) (Supplementary Fig. 3). Using 2 SSc fibroblast lines and microarray gene expression profiling, we determined that approximatively 43% of the differentially expressed genes detected in SSc dermal fibroblasts at P4 were maintained through the 7-day function assay (Supplementary Fig. 3A,B). Although the differential expression of some of the genes may not have passed statistical significance, there was still a statistically significant correlation between the log2 fold changes of the differentially expressed genes from SSc dermal fibroblasts P4 compared to healthy fibroblasts and SSc dermal fibroblasts P4 after the 7-day assay compared to healthy fibroblasts (Supplementary Fig. 3C). Gene set enrichment analysis confirmed that the published *in vitro* TGFβ signature remained enriched in differentially expressed genes between the SSc dermal fibroblasts P4 after the 7-day assay and the healthy fibroblasts, suggesting that the pathway is still active in the 7-day culture assay (Supplementary Fig. 3D).

### Identification of regulators of ASMA expression in SSc dermal fibroblasts

**Figure 4:**
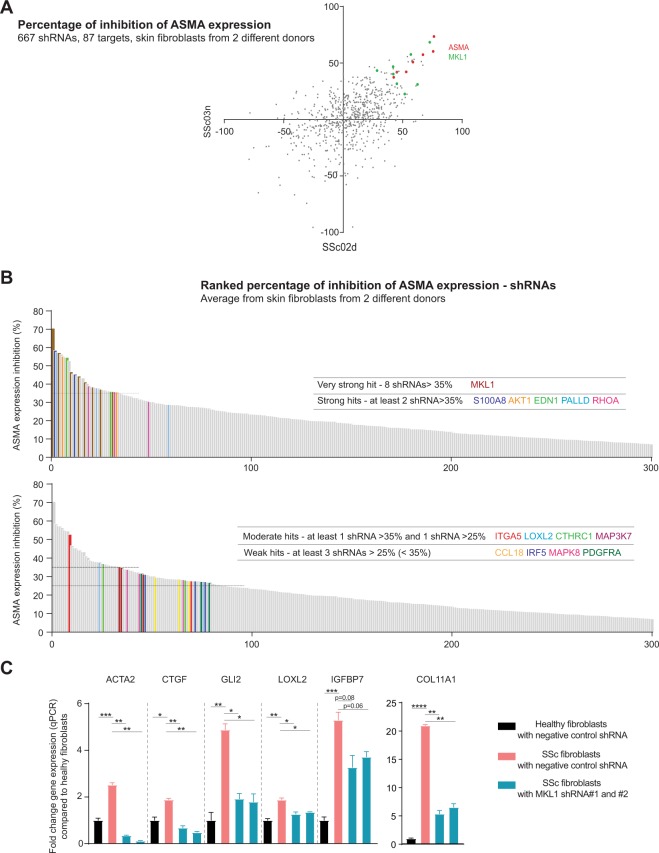
Identification of regulators of ASMA expression in primary SSc dermal fibroblasts. (**A**) Inhibition of ASMA protein expression was determined using ASMA immunostaining after image quantification and normalization with the number of nuclei after transduction of shRNAs into dermal fibroblasts obtained from 2 different SSc donors. ASMA expression level for each shRNA was compared to the ASMA expression level after the negative control GFP shRNA transduction. Plot shows the percentages of ASMA inhibition for the 667 different shRNAs transduced into dermal fibroblasts obtained from 2 different donors. (**B**) Percentage of ASMA inhibition was averaged between the fibroblasts from 2 different donors and ranked from highest average inhibition to lowest inhibition values. (**C**) Quantitative PCR gene expression data of SSc upregulated genes after transduction of negative control shRNA or two different MKL1 shRNAs (cells from 2 healthy donors and 1 SSc donor). Statistical significance was assessed using two tailed Student’s t-test with *p < 0.05, **p < 0.01, ***p < 0.001 and ****p < 0.0001.

After we confirmed that the adenovirus shRNA platform in primary SSc dermal fibroblasts could achieve a robust gene knockdown efficiency and allow the identification of inhibitors of ASMA expression, we wanted to provide biological validation of the assay by testing 667 shRNAs targeting a set of 87 genes enriched for genes predicted to regulate myofibroblast biology (Supplementary Table 3). The 667 shRNAs were tested in dermal fibroblasts obtained from two different SSc donors. The level of ASMA expression was measured by immunofluorescent staining and the percentage of inhibition of ASMA expression was reported for each shRNA tested with dermal fibroblasts from the two SSc donors (Fig. 4A). Multiple shRNAs targeting *ASMA* and *MKL1* showed the strongest inhibition of ASMA in dermal fibroblasts from 2 different SSc donors, confirming the ability of the assay to identify strong regulators of the myofibroblast phenotype (Fig. 4A). The percentage of inhibition of ASMA expression for all the shRNAs was averaged from the values obtained from the two SSc donors and ranked based on the percentage of ASMA expression inhibition (Fig. 4B). *MKL1* shRNA was identified as a very strong hit with 8 different shRNAs yielding to more than 35% inhibition. *S100A8*, *AKT1*, *EDN1*, *PALLD*, *RHOA* shRNAs were considered as strong hits with at least 2 different shRNAs leading to more than 35% inhibition. *ITGA5*, *LOXL2*, *CTHRC1*, *MAP3K7* were considered as moderate hits with at least 1 shRNA with more than 35% inhibition and 1 shRNA with less than 30% inhibition, but more than 25% inhibition. Finally, *CCL18*, *IRF5*, *MAPK8*, *PDGFRA* were considered as weak hits with at least 3 shRNAS with more than 25% inhibition, but less than 35% inhibition.

In order to confirm the modulation of the SSc myofibroblast phenotype, we investigated the effect of *MKL1* knockdown on the expression of other fibroblast SSc-upregulated genes in our gene signature and associated with fibrosis. We confirmed that 2 different shRNAs targeting *MKL1* were able to decrease in SSc fibroblasts the expression of *ACTA2* (encoding for ASMA), extracellular matrix associated genes (*COL11A1*, *LOXL2*, *CTGF*), a known myofibroblast transcription factor (*GLI2*) and a known profibrotic secreted factor (*IGFBP7*) (Fig. 4C). Note that, as reported above, the low FBS assay condition affected the SSc transcriptional signature, such that the expression fold changes between the SSc fibroblast cells and healthy cells is lower during the low FBS assay condition than what we measured from the cells after passage 0 and passage 4 in Fig. 1D.

Together, these results demonstrate our RNAi assay with carefully phenotyped primary dermal fibroblasts from SSc donors can be used to identify regulators of the myofibroblast phenotype of SSc dermal fibroblasts.

## Discussion

Primary dermal fibroblasts from patients affected with skin fibrosis have been considered to be an attractive system to explore the potential of anti-fibrotic drugs^27^. It is commonly thought that these primary fibroblasts could be used up to 10 cell culture passage to avoid senescence effect or to maintain their abnormal collagen production^27,34^. Our results provide the first comprehensive transcriptomic characterization of freshly isolated vs *in vitro* expanded primary SSc dermal fibroblasts after approximately four cell culture passages that correspond to approximately 10 cell doublings (30–40 million cells). Fibroblasts were obtained from patients diagnosed with early diffuse cutaneous SSc with less than 18 months since the first non-Raynaud symptom which is thought to be a disease stage where fibrosis disease modifiers could have an impact. Our results highlight that these primary cells maintain about 77% of their transcriptional disease signature for at least four *in vitro* culture passages. Interestingly, we also confirmed that this disease fibroblast signature is detected in fibroblasts obtained from both clinically affected skin and non-affected skin, consistent with a previous reports of skin biopsy and fibroblast analysis^7,9,16,28,29^. It is important to note that low serum culture conditions often used in typical fibroblast functional assays may further affect the maintenance of SSc disease fibroblast transcriptional phenotype, as we observed a further drift of the SSc fibroblast signature after a 7-day assay in low serum medium culture condition. Overall, while cultured primary SSc dermal fibroblasts do not retain completely the disease transcriptional signature, they still retain a large part of it as well as additional disease phenotypes, such as a higher expression of ASMA and an abnormal cell impedance. Our data support the recommendation to monitor the SSc fibroblast disease transcriptional signature to confirm that the primary cells used in SSc functional assays retain their disease phenotype. Our results suggest avoiding the use of cells after 10 cell doublings as they start to lose their disease transcriptional signature. Note that our results also highlight that ASMA expression started to increase in healthy fibroblasts at passage 4/5, such that high ASMA expression after these passages may be due to cell culture conditions rather than the original disease phenotype. It is possible that this ASMA expression increase is due to the culture on standard tissue culture plastic ware, which provides a high stiffness substrate that is thought to promote myofibroblast differentiation^35^. Together these findings supported the use of low cell passaged and carefully-phenotyped primary cells for functional investigations to identify regulators of the SSc dermal fibroblast disease phenotypes.

One limitation of our study was the limited number of patients that could be enrolled in that study (n = 8), given the low frequency of the disease and that we restricted the study to early diffuse disease. Nevertheless, the transcriptional signature observed in disease fibroblasts was consistent and robust enough such that it could be clearly detected by sample clustering and passed statistical significance (almost 1,000 genes with fold change >1.5 and FDR-adjusted p-value < 0.01, from cells isolated from 6 SSc patient enrolled at the time of the analysis). Importantly, the results are similar to previous studies, including the detection of a TGFβ signature (Fig. 1E), further providing additional confidence in the data collected. As for functional assays, to our knowledge, this is the first original study describing multiple functional readouts from at least fibroblasts from 5 different SSc patients given the difficulty to enroll these patients and obtain skin biopsies (ASMA expression (n = 7), cell impedance (n = 5), shRNA performance with ASMA (n = 6)). Our results were reproducible enough that they passed statistical significance; however, the robustness of these phenotypes across patients remained to be further observed from future independent studies from other groups, given our relatively limited access to patients.

Using an adenovirus RNAi platform^36^, we confirmed that primary SSc dermal fibroblasts could be used a system to identify regulators of their myofibroblast phenotype using ASMA expression as a readout. The assay performance was robust with cells obtained from 6 different SSc donors. Beside technical validation, the biological validation of that assay was also achieved as we successfully detected shRNAs directed at genes that are currently considered as promising anti-fibrotic targets among the strongest inhibitors of ASMA expression in dermal fibroblasts obtained from 2 different SSc donors. *MKL1* and *RHOA* are key regulators of the actin cytoskeleton and are being studied intensively as a potential avenue for anti-fibrotic drug targets^22,37–39^. In particular, MRTF-A protein, encoded by *MKL1*, was shown to be increased in fibroblasts of SSc skin tissue and *MKL1* knockdown was known to decrease contractility and Type I collagen production^22^. Another strong inhibitor of ASMA expression in SSc dermal fibroblasts identified in our assay was LOXL2, which is a lysyl oxidase that promotes the cross-linking of collagen and elastin^40^. Its role in the regulation of the extracellular matrix has been thought to be critical for the development and maintenance of fibrosis, as LOXL2 inhibitors have shown benefit in liver and lung fibrosis mouse models and serum LOXL2 levels have been associated with poor prognosis in patients suffering from idiopathic pulmonary fibrosis (IPF)^41–43^. However, a clinical trial that tested an anti-LOXL2 mAb in IPF failed to show efficacy^44^. Lysyl oxidase activity was shown to be increased in SSc skin and lysyl oxidase serum concentrations correlated with the mRSS clinical score, which evaluates patient’s skin thickness^45,46^. It remains to be investigated whether a strategy to block LOXL2 may be beneficial in SSc skin fibrosis.

Among the 87 genes we tested, it is noteworthy that the shRNAs targeting the SMAD family were not identified as the strongest regulators of the ASMA expression in SSc dermal fibroblasts: 1 *SMAD2* shRNA led to more than 35% inhibition of ASMA expression; 1 *SMAD3* shRNA and 2 *SMAD4* shRNAs led to more than 25%, but less than 35% inhibition of ASMA expression. This was somewhat surprising, given that i) the TGFβ pathway is considered to be a major driver of myofibroblast differentiation and ii) we confirmed the enrichment of the TGFβ gene signature in the SSc dermal fibroblast transcriptomes. This result may be due to redundant role of *SMAD2* and *SMAD3* and the fact that our assay did not include the addition of exogenous recombinant TGFβ, which would have introduced a bias toward the identification of modulators of the TGFβ pathway as the strongest hits.

Several shRNAs targeting *IRF5* showed a weak inhibitory effect on the myofibroblast phenotype of the primary SSc dermal fibroblasts. Genetic polymorphisms in *IRF5* have been associated with multiple auto-immune diseases, including SSc^47^. Although one may speculate that this result may have been linked to the use of a viral vector to deliver the shRNAs, there are several reports suggesting that IRF5 may be involved in myofibroblast or fibroblast biology: fibroblasts from *Irf5* KO mice showed a decrease production of collagen and other ECM components, which may contribute to the protection against disease seen with *Irf5* deficiency in the bleomycin skin mouse model and with the Irf5 inhibition in the tight skin mice^48,49^. Our results combined with these reports suggest that additional studies are needed to confirm the role of *IRF5* in the SSc dermal fibroblast disease phenotypes using non-viral inhibitor strategies.

As discussed above, *LOXL2*, *RHOA and MKL1* have been already extensively studied for their role in fibrosis. The integrin α5, encoded by *ITGA5*, has not yet been examined closely for its role on skin fibrosis. It is known as a receptor of fibronectin, but has not been shown to promote TGFβ activation like some αv integrin heterodimers. The increase α5 expression is associated with poor prognosis of several types of solid tumors and functional studies have demonstrated that α5 promotes invasiness, metastasis and angiogenesis^50^. However, volociximab, an anti-α5β1 blocking antibody, has yet to show efficacy in the treatment of solid tumors^50^. In the context of fibrosis, there are limited studies exploring the role of α5 integrin biology. *In vitro* studies have shown that α5 can be upregulated by CTGF and TGFβ and that blockade α5β1 seems to inhibit TGFβ-induced fibronectin matrix deposition from airway smooth muscle cells^51–53^. Dioscin, a molecule shown to have anti-fibrotic activity in liver and lung fibrosis mouse models, has been speculated to act through α5 integrin^54^. Thus, studies with a more specific inhibitor will be needed to definitely establish the role of α5 in the development of fibrosis. Our results combined to the reports linking α5 to fibronectin, TGFβ and CTGF biology would warrant further translational and *in vivo* investigations of the role of α5 in SSc skin fibrosis.

The phenotyping of the SSc dermal fibroblasts combined with the RNAi high throughput assay we described offer a unique opportunity to investigate regulators of SSc fibroblast biology. There are still very limited studies using primary disease patient cells that report the effect of potential SSc fibroblast disease-modifiers. These studies are often independent, which makes comparisons between different SSc fibroblast disease-modifiers difficult, as they are rarely compared directly to each other’s in a same study. In addition, there are only a few reports of screens to identify potential anti-fibrotic molecules and these studies used either cell lines or rat primary hepatic stellate cells combined with stimulation with TGFβ or other profibrotic agonists^22,55–57^. Our system is the first successful report of the use of primary human disease fibroblasts for high throughput screening which will allow a comprehensive identification of regulators of dermal fibroblast disease phenotypes and provide the opportunity to compare and rank potential regulators to prioritize further efforts on the most potent pathways. While we believe our primary screen to be a robust approach to identify regulators of the SSc myofibroblast phenotype, these results relied mainly on the measurement of ASMA expression levels and validation by a set of SSc myofibroblast gene markers. Additional efforts will be needed to confirm the effects of hits on other readouts of SSc myofibroblast biology, such as contractility and a production of extra-cellular matrix components and ultimately disease mouse models. Together our results provide a comprehensive characterization of primary SSc dermal fibroblasts to highlight both the potentials and limitations of this platform to explore SSc fibroblast disease biology. Our study is the first report of the use of phenotypically characterized primary human patients’ cells for identification of modifiers through screening. We developed a robust high throughput RNAi screen that can identify regulators of the myofibroblast phenotype of primary SSc cells, confirming the relevance of known potential targets already considered for skin fibrosis as well as several potential genes of interest that could be the focus of additional investigations.

## Patients and Methods

### Patient population and skin biopsy collection

The skin biopsies were performed after informed consent and according to European and Dutch regulation, following approval by the medical ethical committee of the Leiden University Medical Center, Leiden, The Netherlands (protocol reference P13.182, NL46199.058.13). Skin biopsies from healthy subjects were collected following informed consent by subjects from remaining skin after corrective resection surgery. Eight SSc patients enrolled the study and were diagnosed with diffuse cutaneous SSc with less than 18 months since the first non-Raynaud symptom. Demographics and skin biopsy locations for all healthy or SSc subjects as well as disease characteristics and treatment information for each SSc patients are available in Table 1. Most patients were positive for anti-topoisomerase antibodies and had not received immunosuppressive therapy. When possible, biopsies were taken from clinically affected and non-affected skin. Skin biopsies were used to isolate primary fibroblasts. Note that all the assays could not be performed for all the fibroblast cultures from all the donors; information on what SSc fibroblasts were evaluated for each assay is reported in Supplementary Table 1.

### Dermal fibroblast isolation and culture

Skin biopsies were minced in small pieces and treated with 2.5 U/mL dispase II (Roche Diagnostics) overnight at 4 °C to allow separation of the epidermis and dermis. Fibroblasts were then extracted from the dermis compartment after incubation for 2 hours at 37 °C in 2.5 U/mL dispase II and 750 U/mL collagenase II (Gibco). After centrifugation, cells were resuspended in DMEM with 10% fetal bovine serum (to support cell survival and proliferation) and 1% penicillin-streptomycin. Cells (designated passage 0) were plated in tissue culture-treated plates or flasks and incubated at 5% CO2 in a humidified atmosphere at 37 °C. When reaching an approximate 70% confluence, cells were trypsinized using 0.05% trypsin-EDTA for 5 minutes at 37 °C and seeded at 1,000 cells/cm2 in 96-well plates or in tissue culture flasks up to passage four (P4), which corresponds to approximately 10 cell doublings from the freshly isolated cells.

### RNA extraction and micro-array data collection

Isolation of RNA from cultured cells was performed by addition of RLT buffer (RNeasy kit, Qiagen) with 1% β-mercaptoethanol. Isolation of total RNA from the cell lysates was performed using the RNeasy Plus 96 or RNeasy Mini kit (Qiagen) and from frozen biopsies using the RNeasy fibrous kit (Qiagen). Total RNA samples were quantified on the Nanodrop 2000 (Thermo Scientific). RNA quality was assessed using TapeStation 2200 (Agilent Technologies). Samples demonstrating low total RNA concentrations were processed with RNA clean & concentrator kit (Cat. R1080, Zymo Research).

Sample labeling and hybridization were performed using The GeneChip™ HT HG-U133+ PM 96-Array Plate (Affymetrix Inc). Washing and staining of the hybridized arrays were completed as described in the GeneChip Expression analysis technical manual for HT plate arrays using the Genechip Array Station (Affymetrix Inc). The processed GeneChip plate arrays were scanned using GeneTitan scanner (Affymetrix Inc). RNA integrity was assessed using the HT RNA reagent kit (Part number 760410, Caliper Life Sciences) using a LabChip GX (PerkinElmer). RNA samples with a RQS score of >8.0 were considered high quality for downstream microarray processing. All microarray transcript profiling data are deposited in the Gene Expression Omnibus (GEO) database (GSE# TBD).

### Analysis of Affymetrix data

Affymetrix scans were subject to quality control (QC) measures, including visual inspection of the distribution of raw signal intensities and assessment of RNA degradation, relative log expression (RLE), and normalized unscaled standard error (NUSE). CEL files were subjected to GC-content-based Robust Multi-array Average (GCRMA) normalization^58–60^. Expression levels were log (base 2) transformed. All calculations and analyses were carried out using R and Bioconductor computational tools. For the fibroblast dataset, a strong batch effect was observed as the data came from two different profiling experiments. Nine replicate samples were included in both experiments to control for batch effect and after batch correction. ComBat (R version 3.3.0, Bioconductor sva package version 3.20.0) was used to adjust for batch effects, which uses empirical Bayes frameworks for adjusting data for batch effects, by inputting the GCRMA normalized data^33,61^. Disease status was included as the covariate of interest.

Analyses were applied to discover genes that were differentially expressed (DEGs). Based on principal component analysis, a few samples were considered to be outliers and removed for downstream analyses (outlier in the biopsy dataset: SSc07_A, outliers in the fibroblast dataset: HS10_P1, SSC04_A_P0 and SSc04_NA_P0). The 9 replicate samples present also in the second fibroblast profiling experiment and that were included to support technical batch effect evaluation and correction were also removed for downstream gene expression analysis. To identify differentially expressed genes between groups of samples, we applied the Linear Models for Microarray (LIMMA) data analysis and paired LIMMA when analyzing difference between affected vs non-affected samples or passage 4 vs passage 0 and passage 1 samples as these were a collection of paired samples originating from the same patient^62^. All p-values for each probe were adjusted using False Discovery Rate (FDR) using the Benjamini and Hochberg method^63^. Genes that exhibited a log-odds score (lods, Limma B statistics) greater than zero, absolute fold change greater than 1.5, FDR adjusted p-value less than 0.01 and average normalized intensity greater than four were considered significantly different. Z-scores and heatmaps were generated using Gene-E software (Broad Institute, Boston, MA, version 3.0.215). Reactome pathway enrichment analyses were performed using EnrichR^64,65^. Gene Set Enrichment Analysis (GSEA) was performed by using GSEA desktop client (Broad Institute, Boston, MA, version 2.2.4).

### ASMA expression levels

To determine levels of ASMA expression at various culture passages, SSc- and healthy donor–derived dermal fibroblasts were seeded in 384-well plates at 2,000 cells/well in 10% FBS-containing DMEM and subsequently fixed with 4% formaldehyde in PBS at day 7 for imaging. For the determination of ASMA expression, fixed dermal fibroblasts were incubated in blocking buffer (PBS containing 0.03 g/mL BSA and 0.2% (vol/vol) Triton X-100) for one hour at room temperature. Cells were then incubated with a monoclonal anti-αSMA antibody (0.8 µg/mL, Cat. AB7817, Abcam) for one hour at room temperature, washed with PBS and incubated with an Alexa Fluor® 546-conjugated donkey anti-mouse secondary antibody (8 µg/mL, Cat. A10036, Life technologies) for one hour at room temperature. Plates were then washed with PBS, incubate with 4′,6-diamidino-2-fenylindool (DAPI) and imaged on a IN Cell 2200 instrument (GE Healthcare). Expression was quantified using algorithms developed with the IN Cell developer software (GE Healthcare). ASMA expression is presented as the staining intensity (density; D) multiplied by the stained area (A) and normalized for the number of DAPI-positive nuclei (DxA/nuclei).

### Adenoviral transduction of dermal fibroblasts

Adenoviruses were produced using PerC6 cells as described earlier^36^. Dermal fibroblasts were seeded at 2,000 cells/well in 384-well plates and at 8,000 cells/well in 96-well plates in 10% FBS-containing FBS. FBS levels in media were titrated to determine an optimal FBS concentration that supports cell survival while not affecting ASMA expression in presence and absence of TGFβ1 (not shown). Based on the results from the FBS titration, cells were serum-deprived in 0.5% FBS-containing DMEM two days after seeding and subsequently transduced with adenoviral shRNAs at a multiplicity of infection (MOI) of 20 to 40 infectious units (IU) per cell. One day later (Day 4) the adenoviral vectors were removed and cells were refreshed with 0.5% FBS-containing DMEM. Three days later (day 7) supernatants were harvested to measure EDA-FN production and cells were fixed for immunofluorescent ASMA staining or lysed for RNA isolation. EDA-FN levels in dermal fibroblast culture supernatants were determined using the MesoScale Discovery (MSD) platform. Isolated total RNA was used to determine target gene knock-down by real time PCR using gene specific Taqman probes (Thermo Fisher Scientific).

### Dermal fibroblast impedance measurements

Healthy donor and SSc patient-derived dermal fibroblasts were seeded at 8,000 cells/well in 10% FBS-containing DMEM on E-plate CardioECR 48-well plates (ACEA Biosciences). The next day cells were serum-deprived in 0.5% FBS-containing DMEM and impedance, presented as Cell Index, was measured over a 7-day period on an xCELLigence® RTCA CardioECR instrument (ACEA Biosciences).

## Supplementary information

Supplementary information

Supp Table 1

Supp Table 2

supp Table 3

Supp Table 4

Supp Table 5
