# Supplementary material for: Identification of regulators of the myofibroblast phenotype of primary dermal fibroblasts from early diffuse systemic sclerosis patients
Source: Sci Rep. 2019 Mar 14;9:4521. doi: 10.1038/s41598-019-41153-w (PMC6418101; doi:10.1038/s41598-019-41153-w)
Supplement: Supplementary file 1 — Supplementary information [file 41598_2019_41153_MOESM1_ESM.pdf]

Identification of regulators of the myofibroblast phenotype of primary dermal fibroblasts from early diffuse systemic sclerosis patients

Loubna Chadli [1], Britt Sotthewes [1], Kejie Li [2], Stefan N. Andersen [1], Ellen Cahir-McFarland [2], Marc Cheung [1], Patrick Cullen [2], Annemarie Dorjée [3], Jeska K. de Vries-Bouwstra [3], Tom W. J. Huizinga [3], David F. Fischer [1], Jeroen DeGroot [1], Joanne L. Viney [2], Timothy S. Zheng [2], Jamil Aarbiou*[,1], Agnes Gardet*[,2] (*co-last, co-corresponding)

[1] Charles River Nederland BV, Leiden, The Netherlands

[2] Biogen, Cambridge, MA, USA

[3] Department of Rheumatology, Leiden University Medical Center, Leiden, The Netherlands

Correspondence should be addressed to agnes.gardet@biogen.com and Jamil.Aarbiou@crl.com

Supplementary Figure 1 – Transcriptomic analyses of skin biopsies from SSc and healthy donors who provided skin biopsies for the isolation of dermal fibroblasts.

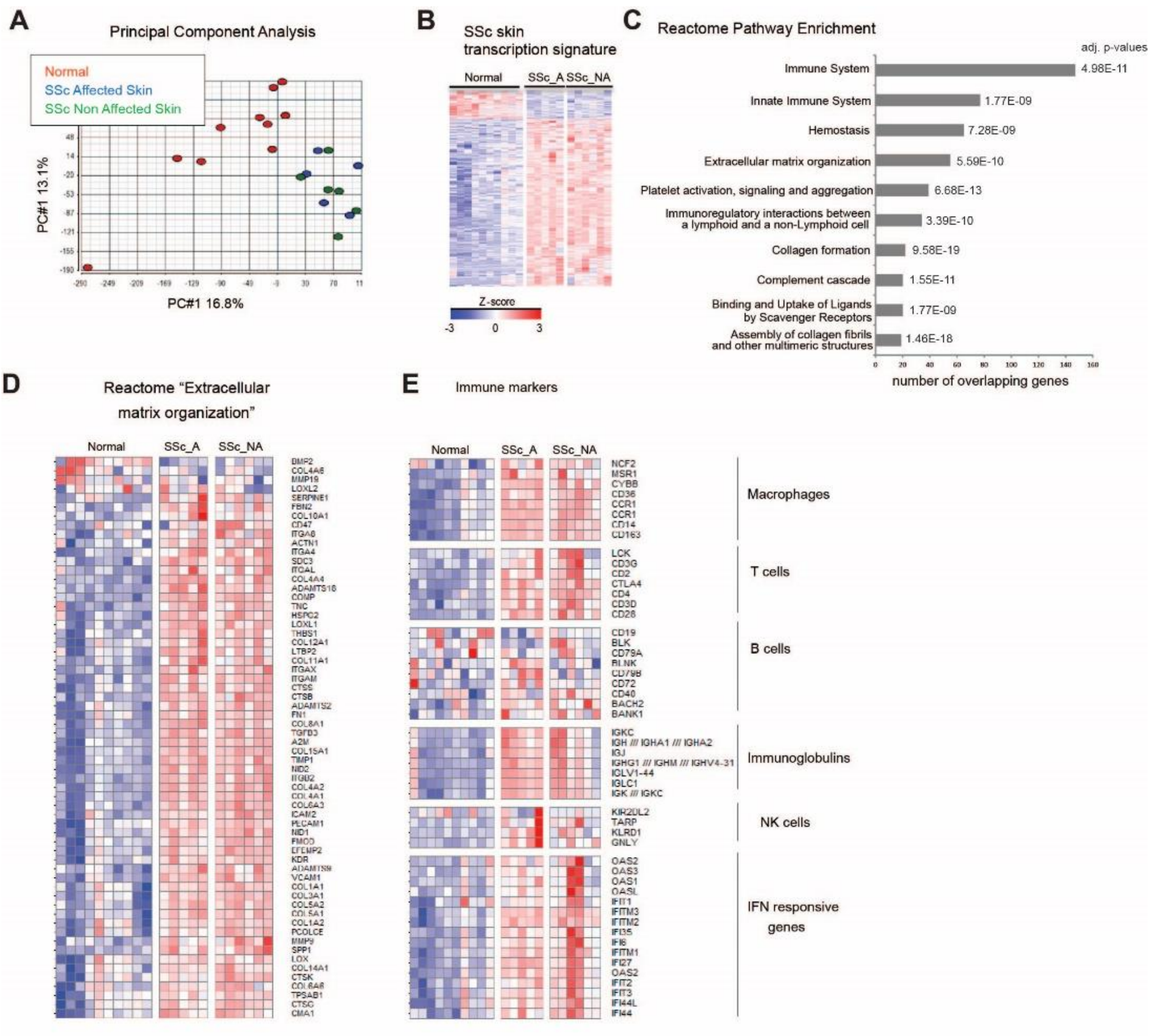

Microarray data from skin biopsies obtained from 6 SSc patients and 10 healthy donors were analyzed. (A) Principal Component Analysis. (B) Z-score heatmap showing expression profile of the differentially expressed probes between SSc and healthy skin biopsies. (D) Reactome pathway enrichment analysis of the SSc skin disease signature. (D and E) Z-score heatmaps showing expression profiles of the differentially expressed probes between SSc and healthy skin biopsies that encodes for proteins involved in the extracellular matrix organization (D) or immune response (E).

Supplementary Figure 2. Batch correction of the dermal fibroblast microarray datasets

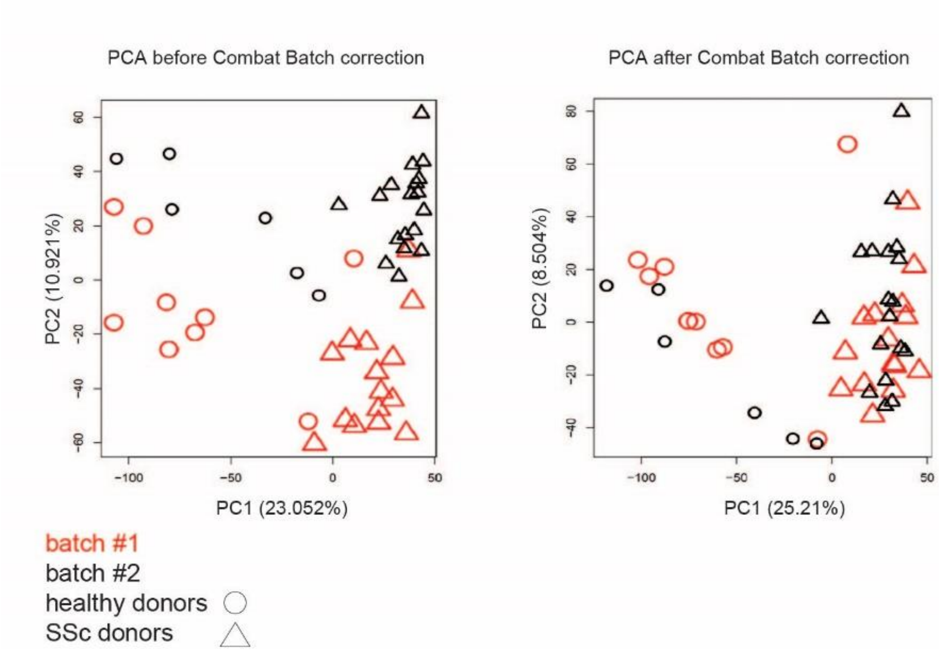

Microarray gene expression data from dermal fibroblasts were collected in two separate batches. Before Combat batch correction, principal component analysis (PCA) shows that samples tend to separate along the principal component #2 based on batch number (right panel). After Combat batch correction, PCA shows sample separation based on disease status and not batch number anymore (left panel).

Supplementary Figure 3. Effect of 7-day culture assay condition on SSc fibroblast transcriptional signature.

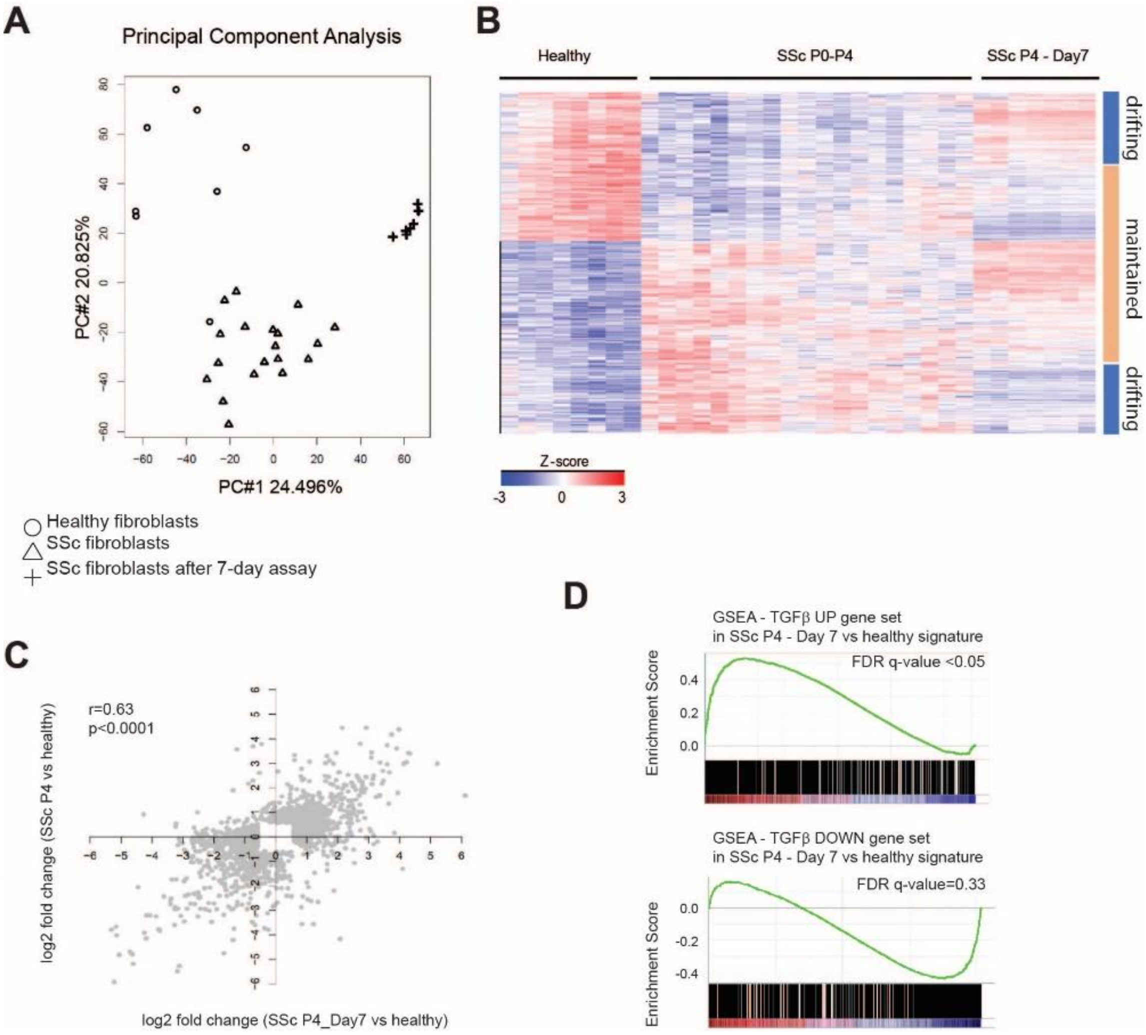

Microarray gene expression data from fibroblasts from 6 SSc patients (isolated from disease affected skin or non-disease affected skin, 6 healthy donors were analyzed and from 2 SSc patients after P4 cell culture passage and 7-day in culture condition ASMA assay. (A) Principal Component Analysis. (B) Z-score heatmap showing the gene expression profiles of the differentially expressed probes between SSc dermal fibroblasts and healthy dermal fibroblasts. (D) Plot showing correlation between the log2 fold changes of the union of the differentially expressed genes from SSc dermal fibroblasts at P4 after 7-day assay compared to healthy fibroblasts and from SSc dermal fibroblasts at P4 vs healthy fibroblasts. (C) Gene Set Enrichment Analysis of a published *in vitro* TGFβ gene signature in the differentially expressed genes between SSc dermal fibroblasts P4 after 7-day assay culture condition and healthy fibroblasts.
